# Clinical Spectrum of Drug Hypersensitivity Reactions in Systemic Mastocytosis: Drug-Induced Anaphylaxis as a Unique Clinical Presentation

**DOI:** 10.3390/medicina62040711

**Published:** 2026-04-08

**Authors:** Eda Aslan, Kasım Okan, Ragıp Fatih Kural, Sinem İnan, Yusuf Özeke, Ümitcan Ateş, Onurcan Yıldırım, Züleyha Galata, Kutay Kırdök, Ecem Ay, Türkan Dizdar Canbaz, Meryem İrem Toksoy Şentürk, Seda Karaaslan Yetemen, Reyhan Gümüşburun, Hatice Serpil Akten, Hasibe Aytaç, Melih Özışık, Asuman Çamyar, Gülhan Demiroğlu, Gökten Bulut, Meryem Demir, Nur Soyer, Fatma Keklik Karadağ, Derya Demir, Mine Hekimgil, Nazan Özsan, Banu Pınar Şarer Yürekli, Emin Karaca, Mehmet Burak Durmaz, Ceyda Tunakan Dalgıç, Ali Kokuludağ, Aytül Zerrin Sin, Emine Nihal Mete Gökmen

**Affiliations:** 1 Department of Internal Medicine, Division of Allergy and Immunology, Ege University Faculty of Medicine, Izmir 35100, Türkiye; edaarslan_91@hotmail.com (E.A.); fatih_k_123@hotmail.com (R.F.K.); ozekeyusuf@gmail.com (Y.Ö.); umitcanates@gmail.com (Ü.A.); zuleyhagalata61@gmail.com (Z.G.); kirdokkutay@gmail.com (K.K.); ecem.ay@live.com (E.A.); turkan88dizdar@gmail.com (T.D.C.); irem.toksoy@yahoo.com (M.İ.T.Ş.); seda.karaaslan95@gmail.com (S.K.Y.); reyhangumusburun@gmail.com (R.G.); dr_ceydat@yahoo.com (C.T.D.); ali.kokuludag@ege.edu.tr (A.K.); aytulsin@yahoo.com (A.Z.S.); 2 Department of Allergy and Immunology, Ordu University Training and Research Hospital, Ordu 52200, Türkiye; kasimokan55@gmail.com; 3 Department of Allergy and Immunology, Izmir City Hospital, Izmir 35540, Türkiye; drsineminan@gmail.com (S.İ.); doktormalih@hotmail.com (M.Ö.); 4 Department of Allergy and Immunology, Osmaniye State Hospital, Osmaniye 80000, Türkiye; onurcanyildirim@hotmail.com; 5 Department of Allergy and Immunology, Dr. Suat Seren Chest Diseases and Surgery Training and Research Hospital, Izmir 35170, Türkiye; aktenhaticeserpil@gmail.com (H.S.A.); hasibeuchkun@hotmail.com (H.A.); 6 Department of Immunology and Allergy Diseases, Izmir Bakircay University Cigli Regional Training and Research Hospital, Izmir 35660, Türkiye; asuerden@yahoo.com; 7 Department of Allergy and Immunology, Izmir Buca Seyfi Demirsoy Training and Research Hospital, Izmir 35390, Türkiye; gulhanbogatekin@gmail.com; 8 Department of Allergy and Immunology, Balıkesir Atatürk City Hospital, Balıkesir 10100, Türkiye; goktenbulut58@gmail.com; 9 Department of Allergy and Immunology, Kartal Dr. Lütfi Kırdar City Hospital, Istanbul 34865, Türkiye; meryem_durgay@hotmail.com; 10 Department of Hematology, Ege University Faculty of Medicine, Izmir 35100, Türkiye; drakadnur@yahoo.com (N.S.); fatma_keklik86@hotmail.com (F.K.K.); 11 Department of Pathology, Ege University Faculty of Medicine, Izmir 35100, Türkiye; dr.derya.demir@gmail.com (D.D.); mine.hekimgil@gmail.com (M.H.); nazanozsan@yahoo.com (N.Ö.); 12 Department of Endocrinology, Ege University Faculty of Medicine, Izmir 35100, Türkiye; bsareryurekli@gmail.com; 13 Department of Medical Genetics, Ege University Faculty of Medicine, Izmir 35100, Türkiye; emin.karaca@ege.edu.tr (E.K.); burak.durmaz@ege.edu.tr (M.B.D.)

**Keywords:** anaphylaxis, β-lactam antibiotics, drug hypersensitivity, nonsteroidal anti-inflammatory drugs, systemic mastocytosis, tryptase

## Abstract

*Background and Objectives*: Systemic mastocytosis (SM) is a clonal mast cell disorder characterized by abnormal mast cell accumulation and activation in multiple organs, leading to mediator-related symptoms, including anaphylaxis. Drug hypersensitivity reactions (DHRs) are a major clinical challenge in SM, but their frequency and characteristics remain undefined. This study aimed to evaluate the frequency of drug allergy, identify high-risk drug groups, investigate reaction characteristics, and examine the relationship between drug reactions, baseline serum tryptase levels, and SM subtypes in patients with SM. *Materials and Methods:* We retrospectively analyzed 34 patients diagnosed with SM between 2009 and 2024 at Ege University Faculty of Medicine. Clinical features, SM subtypes, baseline serum tryptase levels, and DHR characteristics were recorded. Reactions to antibiotics, nonsteroidal anti-inflammatory drugs (NSAIDs), paracetamol, anesthetics, radiocontrast media (RCM), and COVID-19 vaccines were graded using the Ring and Messmer anaphylaxis classification. *Results*: Among 34 patients, the mean age was 48.6 ± 13.3 years, 53% were male, and 10 (29.4%) had DHRs. The most common culprit drugs were NSAIDs (17.6%) and β-lactam antibiotics (14.7%). Anaphylaxis was the predominant reaction, frequently associated with hypotension. In 5 patients (14.7%), drug-induced anaphylaxis was the initial and only manifestation of SM. No hypersensitivity reactions occurred to quinolones, general anesthetics, or COVID-19 vaccines. Median baseline tryptase was 50.25 µg/L (min–max: 8.59–200.00) overall, and 41.85 µg/L (min–max: 19.00–200.00) among those with DHRs. *Conclusions*: Patients with SM are at increased risk of severe DHRs, particularly to NSAIDs and beta-lactam antibiotics. In some patients, drug allergy may be the first and only manifestation of SM. Measurement of baseline serum tryptase is essential in patients with drug-induced anaphylaxis. A comprehensive allergy assessment, including tolerance testing and individualized counseling, is crucial to ensure safe pharmacological management.

## 1. Introduction

Mast cell diseases are a rare group of disorders characterized by abnormally increased mast cell activity and a broad clinical spectrum. Mastocytosis, the most common of these disorders, is defined by the accumulation of clonal mast cells in the skin and/or extracutaneous organs, including the bone marrow, spleen, lymph nodes, liver, and gastrointestinal tract [1,2,3]. It may present as cutaneous mastocytosis, systemic mastocytosis (SM), or, rarely, mast cell sarcoma.

The diagnosis of SM is established according to World Health Organization (WHO) criteria, requiring either one major and one minor criterion, or at least three minor criteria, based on histopathologic, molecular, immunophenotypic, and laboratory findings. The major criterion is the presence of multifocal, dense infiltrates of mast cells (≥15 mast cells per aggregate) detected in bone marrow biopsies or other extracutaneous organs. The minor criteria include: 1-More than 25% of mast cells exhibiting atypical morphology or spindle-shaped appearance; 2-Detection of an activating KIT D816V mutation in bone marrow or other extracutaneous tissues; 3-Expression of aberrant surface markers CD2, CD25, or CD30 on mast cells; 4-Persistently elevated baseline serum tryptase levels exceeding 20 ng/mL in the absence of another myeloid neoplasm [4]. SM is further classified into non-advanced forms including bone marrow mastocytosis (BMM), indolent systemic mastocytosis (ISM), and smoldering systemic mastocytosis (SSM), which are generally associated with normal life expectancy and advanced forms, such as aggressive systemic mastocytosis (ASM), mast cell leukemia, and systemic mastocytosis with an associated hematologic neoplasm (SM-AHN), which are characterized by organ dysfunction and reduced survival [5]. Cutaneous mastocytosis predominantly occurs in children and is limited to the skin, whereas systemic forms are more common in adults and typically involve the bone marrow and other extracutaneous organs [6]. BMM is a rare variant of non-advanced SM, characterized by mast cell infiltration restricted to the bone marrow in the absence of involvement of other organs. ISM is the most prevalent subtype of non-advanced SM and exhibits a variable clinical course. In these patients, mast cells infiltrate the bone marrow or other extracutaneous tissues without causing organ dysfunction. SSM is diagnosed in patients who fulfill the WHO criteria who have ≥2 B-findings without C-findings or SM-AHN, often reflecting a high marrow mast cell burden and increased rates of hepatosplenomegaly and constitutional symptoms [5].

Patients with mastocytosis experience symptoms resulting from both the increased tissue burden of mast cells and the acute or chronic release of mast cell mediators, as well as the production of cytokines and chemokines. Granule-derived mediators such as tryptase and histamine, along with arachidonic acid metabolites including prostaglandins and leukotrienes, bind to their respective receptors and contribute to a wide range of symptoms, including flushing, diarrhea, gastroesophageal reflux, dizziness, fatigue, headache, hypotension, and anaphylaxis [1,2].

Anaphylaxis is a potentially life-threatening clinical manifestation of SM. Although the prevalence of atopy among individuals with mastocytosis does not significantly differ from that in the general population, the presence of allergic comorbidities in these patients is frequently associated with increased symptom burden and disease severity. Severe anaphylactic reactions, including anaphylactic shock, may occur following Hymenoptera stings or during venom immunotherapy, and in some cases, mastocytosis may clinically present as idiopathic anaphylaxis [7].

However, anaphylaxis and mast cell activation in SM are not limited to venom-related triggers. Mast cells may also be activated by a wide range of pharmacologic agents, leading to drug hypersensitivity reactions (DHRs) and related complications. Commonly implicated drugs include nonsteroidal anti-inflammatory drugs (NSAIDs), antibiotics (particularly quinolones and vancomycin), opioid analgesics, radiocontrast media (RCM), general and local anesthetics, and vaccines. In mast cell diseases, DHRs may occur through both classical IgE-mediated mechanisms and IgE-independent pathways, notably involving activation of the Mas-related G-protein-coupled receptor X2 (MRGPRX2) [8].

Despite the recognized clinical relevance of drug-induced reactions, data regarding the prevalence of DHRs in SM, their distribution across disease subtypes, and associated risk factors remain limited. As a result, pharmacologic management in patients with SM is often challenging. The present study aims to increase awareness and to provide further insight into the frequency, severity, and clinical characteristics of DHRs in patients with SM.

## 2. Materials and Methods

### 2.1. Study Design and Patients

This retrospective observational study evaluated DHRs in adult patients diagnosed with SM. The primary outcome of the study was defined as the occurrence of any DHR in patients with SM. A secondary objective was to identify patients in whom drug-induced anaphylaxis represented the first clinical manifestation leading to the diagnosis of SM.

All consecutive adult patients (≥18 years) with SM who were followed between January 2009 and December 2024 at the Departments of Allergy–Immunology and Hematology, Ege University Faculty of Medicine, were screened for eligibility. A total of 34 patients were identified, and all were included in the final analysis. No patients were excluded due to incomplete medical records or missing clinical data.

Given the retrospective observational design of the study, no formal sample-size calculation or power analysis was performed. The sample size was determined by the number of eligible patients with SM who were followed at our center during the study period.

Hypersensitivity reactions to antibiotics, analgesic agents (NSAIDs and paracetamol), RCM, general and local anesthetics, and COVID-19 vaccines (BNT162b2 [Pfizer–BioNTech, Mainz, Germany] or CoronaVac [Sinovac, Beijing, China] ) were included in the analysis. The diagnosis and subclassification of SM were established according to the 2016 WHO criteria, based on bone marrow biopsy findings [4].

The study protocol was approved by the Ege University Medical Research Ethics Committee (Approval Date: 5 March 2026; Approval No: 26-3T/91).

### 2.2. Data Collection and Clinical Assessment

Patient data were obtained from electronic medical records and included demographic characteristics (age and sex), SM subtype, baseline laboratory parameters, and clinical characteristics of DHRs.

Baseline laboratory evaluations comprised serum tryptase levels, total immunoglobulin E (IgE) concentrations and in vitro test (specific IgE testing for penicillin determinants) measured during the patients’ stable clinical period. Serum tryptase values were recorded as baseline levels prior to hypersensitivity reactions whenever available.

DHRs were classified according to clinical phenotype as anaphylaxis, urticaria, or angioedema. The timing of reactions in relation to drug exposure was recorded and categorized as immediate reactions (occurring within the first hour after exposure) or delayed reactions (occurring after the first hour). The severity of anaphylactic reactions was graded according to the Ring and Messmer classification [9].

### 2.3. Drug Allergy Work-Up

The diagnosis of drug hypersensitivity was established through a comprehensive allergological evaluation, including a detailed clinical history, physical examination, and, when clinically indicated, and skin prick tests (SPTs), intradermal tests (IDTs), and/or oral drug provocation tests (DPTs) performed in a controlled clinical setting. All diagnostic procedures were performed by experienced allergy specialists in facilities equipped with emergency resuscitation equipment to ensure patient safety. In patients with a history of severe anaphylaxis, particularly those with high-grade reactions (Grade 3–4), drug provocation testing was generally avoided due to safety concerns, especially when tolerated alternative medications were available. In such cases, the diagnosis was primarily based on detailed clinical history and medical record documentation. When considered clinically appropriate and safe, skin tests and DPT with alternative medications were performed in accordance with the recommendations of the European Academy of Allergy and Clinical Immunology (EAACI).

#### 2.3.1. Skin Tests

Skin tests were performed on the volar surface of the forearm. Prior to testing, the skin was cleaned with alcohol. Histamine (10 mg/mL) was used as the positive control and normal saline as the negative control. Skin test results were evaluated after 20 min, and a wheal diameter ≥3 mm compared with the negative control was considered positive.

Determinants of Allergic Penicillin (DAP) tests, including penicillin minor and major determinants, clavulanic acid, and amoxicillin (Diater Laboratories, Madrid, Spain), along with benzyl penicillin (penicillin G), cefuroxime, ceftriaxone, the culprit cephalosporins, and ampicillin-sulbactam, were used for SPT and IDT. All drugs and reagents used in the study were commercially available pharmaceutical preparations obtained from standard manufacturers and were used in accordance with routine clinical practice.

For penicillin G, SPTs and IDTs were performed at a maximum concentration of 10,000 U/mL (1:1). IDT was initiated at a 1:1000 dilution and subsequently performed at 1:100, 1:10, and 1:1 dilutions. SPTs and IDTs for ampicillin–sulbactam were performed up to a maximum concentration of 25 mg/mL (1:1). IDT was initiated at a 1:1000 dilution and continued at 1:100, 1:10, and 1:1 (25 mg/mL) dilutions. Cefuroxime and ceftriaxone were reconstituted by dissolving 1 g of the drug in 10 mL of normal saline, yielding a SPT concentration of 100 mg/mL (1:1). IDT was initiated at a 1:1000 dilution of the maximum concentration and subsequently performed at 1:100 and 1:10 dilutions.

For fentanyl (0.05 mg/mL) and propofol (10 mg/mL), SPTs were performed at a 1:1 concentration, and IDT was initiated at a 1:1000 dilution followed by 1:100 and 1:10 concentrations. For tramadol, the SPT was performed at a concentration of 1 mg/mL, and IDT was initiated at a 1:100 dilution and completed at a maximum concentration of 1:10. For rocuronium, the SPT was performed at a concentration of 10 mg/mL (1:1), and IDT was initiated at a 1:20,000 dilution and subsequently performed at 1:2000 and a maximum concentration of 1:200 (0.05 mg/mL).

#### 2.3.2. Drug Provocation Tests

Single-blind, placebo-controlled DPTs were performed in patients with a history of NSAID- or antibiotic-related drug reactions using appropriate alternative medications, taking into account the severity of the previous reaction and the drugs tolerated by the patient. In patients with a history of reactions to non-selective COX-1-inhibiting NSAIDs, meloxicam (7.5 mg) and, when there was no prior reaction history, paracetamol (500 mg) was preferred for oral provocation testing.

In patients with a history of reactions to amoxicillin and/or ampicillin, oral provocation tests were primarily performed with non-β-lactam antibiotics, including ciprofloxacin (500 mg), levofloxacin (500 mg), clarithromycin (500 mg), clindamycin (300 mg), and metronidazole (500 mg), considering the severity of the reaction and the clinical necessity of antibiotic use. When provocation with a β-lactam antibiotic was clinically indicated, ceftriaxone (1 g, administered intramuscularly) and cefuroxime (500 mg, administered orally) were used in patients with a history of amoxicillin hypersensitivity.

DPTs were conducted using a graded dosing protocol. The total therapeutic dose was divided into two administrations, with a two-hour observation interval between doses.

### 2.4. Statistical Analysis

Statistical analyses were performed using IBM SPSS Statistics, version 25.0 (IBM Corp., Armonk, NY, USA). The distribution of continuous variables was assessed using the Shapiro–Wilk test. Variables with normal distribution are presented as mean ± standard deviation (SD), whereas variables that did not follow a normal distribution are reported as median (minimum–maximum).

Comparisons between groups were performed according to the distribution of the variables using parametric or nonparametric tests. For continuous variables with normal distribution, the independent-samples *t*-test was used, while the Mann–Whitney U test was applied for variables that did not meet the assumption of normality. Categorical variables are presented as counts and percentages and were compared using the chi-square test or Fisher’s exact test, as appropriate. The Fisher–Freeman–Halton exact test was used for contingency tables with small, expected cell counts.

Baseline serum tryptase levels were analyzed as a continuous variable. Because the distribution of tryptase levels was non-normal, results are presented as median (minimum–maximum), and group comparisons were performed using the Mann–Whitney U test. No grouping or data transformation was applied to tryptase levels.

Statistical comparisons were performed only between independent patient groups. Accordingly, patients with DHR (DHR-positive) and those without DHR (DHR-negative) were analyzed as two independent groups. The overall cohort was presented using descriptive statistics only. A two-tailed *p*-value < 0.05 was considered statistically significant.

### 2.5. Use of Generative Artificial Intelligence

Generative artificial intelligence tools were not used for data generation, data analysis, or interpretation in this study. AI-assisted tools, if any, were utilized solely for language editing and formatting purposes.

## 3. Results

**Figure 1:**
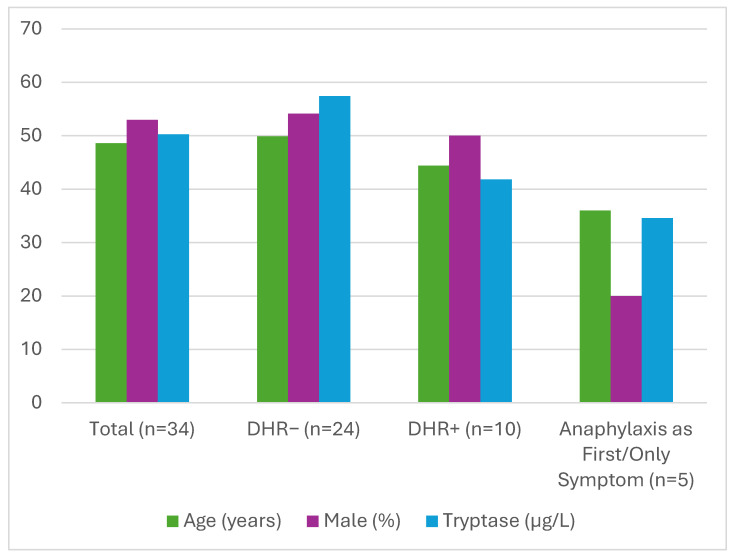
Age, Sex and Serum Tryptase Levels Distribution in Patients with and without Drug Hypersensitivity Reactions, Including the Subgroup Whose First and Only Clinical Manifestation was Drug-Induced Anaphylaxis. Abbreviation: DHR, drug hypersensitivity reaction.

**Figure 2:**
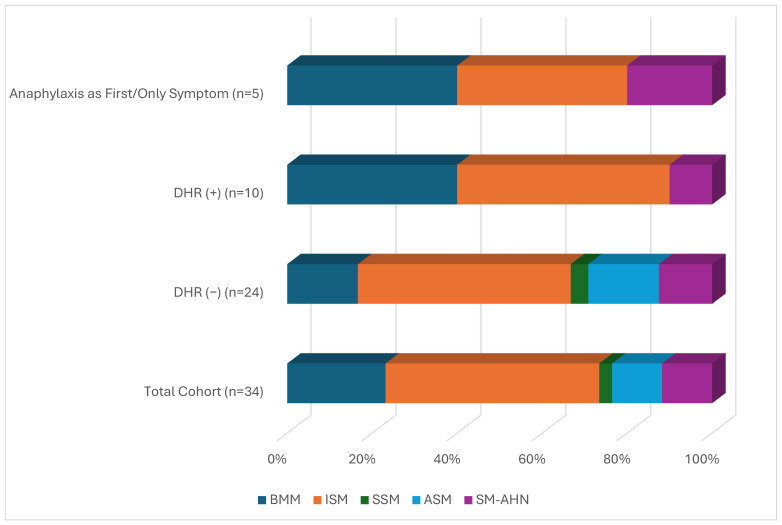
Systemic Mastocytosis Subtype Distribution in Patients with and without Drug Hypersensitivity Reactions, Including the Subgroup Whose First and Only Clinical Manifestation Was Drug-Induced Anaphylaxis. Abbreviations: ASM, aggressive systemic mastocytosis; BMM, bone marrow mastocytosis; DHR, drug hypersensitivity reaction; ISM, indolent systemic mastocytosis; SM-AHN, systemic mastocytosis with associated hematologic neoplasm; SSM, smoldering systemic mastocytosis.

Among the 34 patients with SM, 16 (47%) were female, with a mean age of 48.6 ± 13.3 years. The median baseline serum tryptase level in the overall cohort was 50.25 µg/L (8.59–200.00) (Figure 1). SM subtypes included BMM in 8 patients (23.5%), ISM in 17 (50%), SSM in 1 (3%), ASM in 4 (11.7%), and SM-AHN in 4 patients (11.7%) (Figure 2). In all cases, exposure to the culprit drugs occurred prior to the diagnosis of SM.

DHRs were identified in 10 patients (29.4%), equally distributed between males and females. The median age of patients with DHRs was 42.5 years (30–70), and the median baseline tryptase level was 41.85 µg/L (19.00–200.00). SM subtypes in this group included BMM (*n* = 4), ISM (*n* = 5), SM-AHN (*n* = 1).

Overall, drug hypersensitivity was confirmed in two of the ten patients through positive diagnostic testing, including specific IgE and/or skin testing (Patients 4 and 8). In the remaining patients, the diagnosis of drug hypersensitivity was established based on a compatible clinical history of drug-related anaphylaxis and supporting medical record documentation. In patients with a history of severe systemic reactions, confirmatory testing with the suspected culprit drugs was not performed due to safety considerations. Instead, when considered clinically appropriate and safe, allergological evaluation with alternative medications was carried out to identify tolerated therapeutic options.

In five patients (14.7%), drug-induced anaphylaxis was the first and only clinical manifestation of SM, with no history of spontaneous anaphylaxis. These patients were predominantly female (4/5), with a median age of 36 (30–70) years and a median baseline tryptase level of 34.60 µg/L (20.40–126.00). Reported culprit drugs included amoxicillin (*n* = 3), ampicillin (*n* = 1), ibuprofen (*n* = 1), and flurbiprofen (*n* = 1).

Among the 24 patients without a history of DHRs, 13 (54.1%) were male, with a mean age of 49.9 ± 14.3 years and a median baseline tryptase level of 57.40 µg/L (8.59–200.00). SM subtypes in this group included BMM (*n* = 4), ISM (*n* = 12), SSM (*n* = 1), ASM (*n* = 4), and SM-AHN (*n* = 3).

Comparative analyses between DHR-positive (*n* = 10) and DHR-negative (*n* = 24) patients revealed no significant differences in age, sex distribution, SM subtype, or baseline serum tryptase levels (all *p* > 0.05) (Table 1). Similarly, patients whose first and only manifestation was drug-induced anaphylaxis did not differ significantly from DHR-negative patients with respect to these variables.

### 3.1. Drug Hypersensitivity Reactions to NSAIDs

**Figure 3:**
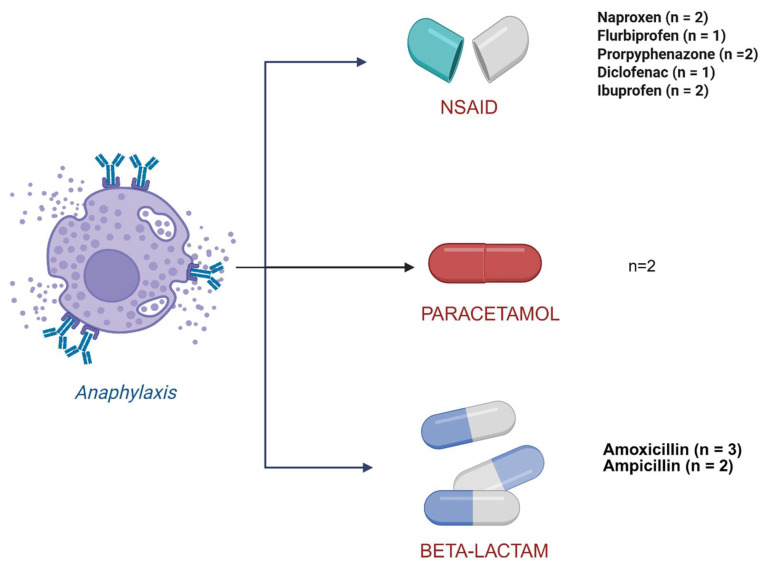
Drugs implicated in anaphylaxis related to NSAIDs, beta-lactams, and paracetamol. Abbreviation: NSAIDs, nonsteroidal anti-inflammatory drugs.

A history of NSAID-induced anaphylaxis was identified in five patients (14.7%). The implicated drugs included naproxen (*n* = 2), flurbiprofen (*n* = 1), propyphenazone (*n* = 2), diclofenac (*n* = 1), and ibuprofen (*n* = 2) (Figure 3). Additionally, two patients (6%) reported urticaria and angioedema following NSAID use (naproxen in one case and indomethacin in the other).

According to the Ring and Messmer classification, one patient experienced grade 4, two grade 3, and two grade 2 anaphylaxis. Hypotension was documented in three patients. Among two patients with paracetamol-induced anaphylaxis, one experienced grade 4 and the other grade 3 anaphylaxis, both accompanied by hypotension. Overall, paracetamol-induced anaphylaxis and urticaria/angioedema were observed in two (5.8%) and one (2.9%) patients, respectively.

**Figure 4:**
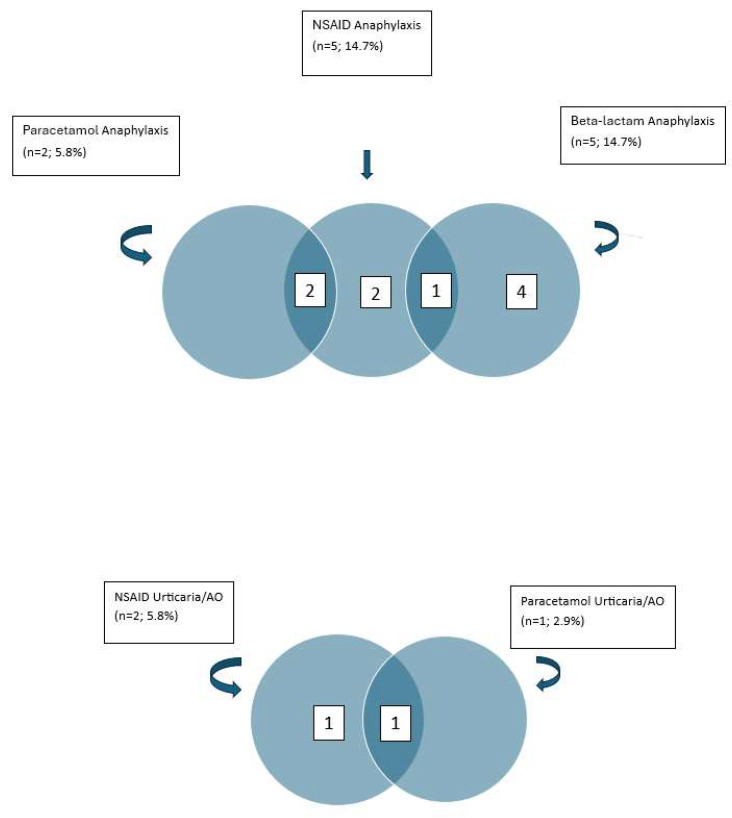
Anaphylaxis and Urticaria/Angioedema. Numbers within the diagrams represent the number of patients in each subgroup and overlap, while arrows indicate the distribution and relationships between different drug hypersensitivity reactions. Abbreviations: NSAID, nonsteroidal anti-inflammatory drug; AO, angioedema.

Notably, both patients with paracetamol-induced anaphylaxis also reported reactions to other NSAIDs (propyphenazone in one case and naproxen in the other; Figure 4).

The median serum tryptase level in patients with NSAID-related DHRs was 41.85 µg/L (range: 19.00–111.00). Among these patients, three had BMM, and three had ISM.

#### Clinical Characteristics of NSAID-Induced Reactions

NSAID-associated DHRs were observed in five patients and presented with a broad spectrum of clinical manifestations, with symptom onset ranging from 10 min to 4 h after drug intake.

Severe systemic reactions consistent with grade 3–4 anaphylaxis were observed in several patients (patients 1, 2, 5, and 10). These reactions were characterized by cardiovascular and respiratory involvement, including hypotension, dyspnea, tachycardia, and loss of consciousness. Notably, one patient (patient 1) experienced a life-threatening episode with cardiac arrest approximately 15 min after ingestion of a fixed-dose combination of propyphenazone and paracetamol, requiring immediate treatment with epinephrine, antihistamines, corticosteroids, and defibrillation.

Recurrent systemic reactions to different NSAIDs were documented in patients 2 and 10, who experienced multiple episodes following exposure to flurbiprofen, ibuprofen, propyphenazone, and diclofenac, suggesting possible cross-reactivity among NSAIDs.

Moderate systemic reactions compatible with grade 2 anaphylaxis were observed in patients 9 and 10 (after exposure to propyphenazone), presenting with symptoms such as facial flushing, dyspnea, tachycardia, and pruritus shortly after NSAID intake.

In contrast, isolated cutaneous hypersensitivity reactions without systemic involvement were documented in patient 6, presenting as generalized urticaria with genital edema approximately four hours after indomethacin intake and resolving with antihistamine treatment.

During allergological evaluation and follow-up, oral provocation tests with alternative analgesics, including paracetamol and meloxicam, were performed in selected patients (patients 2 and 9) and yielded negative results, indicating tolerance to these agents. In patients with severe or life-threatening reactions (patients 1, 5, and 10), further allergological testing with the suspected NSAIDs was not performed due to safety concerns. In patient 6, additional drug provocation testing was not considered necessary because the patient subsequently tolerated alternative analgesics, including meloxicam, nimesulide, and paracetamol, without adverse reactions.

The detailed clinical characteristics, severity grading, and allergological evaluation results are summarized in Table 2.

### 3.2. Drug Hypersensitivity Reactions to β-Lactam Antibiotics

Anaphylactic reactions to β-lactam antibiotics were reported in five patients (14.7%), triggered by amoxicillin (*n* = 3) and ampicillin (*n* = 2) (Figure 3). None of these patients developed isolated cutaneous reactions. One patient experienced anaphylaxis to both β-lactam antibiotics and NSAIDs (Figure 4).

All β-lactam-related reactions were classified as grade 3 anaphylaxis, and hypotension was present in four patients. The median serum tryptase level in this group was 34.60 µg/L (20.4–200). SM subtypes included ISM (*n* = 3), BMM (*n* = 1), and SM-AHN (*n* = 1).

#### Clinical Characteristics of β-Lactam Antibiotics-Induced Reactions

β-lactam antibiotic-related DHRs were observed in five patients (patients 3, 4, 7, 8, and 10), most commonly following exposure to amoxicillin or ampicillin (Table 2). Symptom onset ranged from 10 min to approximately 6 h after antibiotic administration. All reactions were consistent with grade 3 anaphylaxis and were characterized by respiratory and cardiovascular involvement, including dyspnea, hypotension, syncope, dizziness, and gastrointestinal symptoms. In one patient (patient 3), anaphylaxis developed approximately 6 h after ampicillin administration and required monitoring in the intensive care unit for one day.

Serum specific IgE testing for penicillin determinants was performed in selected patients. Positive results were observed in patients 4 and 8, whereas negative results were obtained in patients 3, 7, and 10.

Additional allergological evaluation with skin testing and/or drug provocation testing using alternative antibiotics was performed in selected patients. In patient 4, further diagnostic evaluation demonstrated a positive DAP test, with SPT positivity to the penicillin major determinant at a 1/1 concentration (9/40 mm) and to penicillin G at a 1/1 concentration (4/10 mm), whereas the negative control (normal saline) yielded a 0 mm response, supporting IgE-mediated hypersensitivity. In patient 7, SPTs and IDTs with cefuroxime, penicillin, ceftriaxone, and ampicillin–sulbactam were negative, and tolerance to alternative antibiotics was confirmed by intramuscular provocation testing with ceftriaxone and oral provocation tests with cefuroxime and levofloxacin. Similarly, oral provocation tests with clarithromycin, metronidazole, and clindamycin were negative in patient 8, while tests with ciprofloxacin and clarithromycin were negative in patients 4 and 10, indicating tolerance to these alternative antimicrobial agents. In patients with severe systemic reactions or a high risk of recurrence (patients 3 and 10), further allergological testing with the suspected culprit β-lactam antibiotics was not performed due to safety considerations.

Overall, these findings demonstrate variable clinical presentations and heterogeneous diagnostic test results in β-lactam-associated hypersensitivity reactions within the cohort.

The detailed clinical characteristics, diagnostic testing results, and tolerated alternative antibiotics are summarized in Table 2.

Among 26 patients who received quinolones, no hypersensitivity reactions were reported.

### 3.3. Drug Hypersensitivity Reactions to Local and General Anesthetics

Among the 28 patients who had received local anesthetics, only 1 patient (3.5%) experienced an anaphylactic reaction with lidocaine. The reaction was classified as grade 2 anaphylaxis and occurred approximately 15 min after drug exposure, presenting with facial flushing, urticaria, and dizziness. The reaction was successfully managed with antihistamine treatment. As the patient was scheduled for general anesthesia, further allergological evaluation was performed. SPTs and IDTs with fentanyl, propofol, tramadol, and rocuronium, as well as a SPT with latex, yielded negative results, indicating tolerance to these agents (Table 2). The patient’s serum tryptase level was 37.5 ng/mL, and she was diagnosed with ISM.

Of 24 patients who underwent general anesthesia prior to being diagnosed with SM and without premedication, no hypersensitivity reactions were observed.

### 3.4. Drug Hypersensitivity Reactions to Radiocontrast Media

In 23 patients who received RCM without premedication before a procedure, anaphylaxis occurred in 1 patient (4.3%). Despite detailed history-taking, the specific agent responsible could not be identified. A systemic reaction compatible with grade 2 anaphylaxis was observed following RCM in one patient (patient 5), presenting with pruritus, urticaria, and vomiting approximately one hour after contrast administration. The reaction was managed with pheniramine treatment. Further allergological evaluation was not performed because the exact contrast agent could not be identified, and there was no subsequent clinical indication for contrast-enhanced imaging (Table 2). This 32-year-old female patient (patient 5) also had a history of anaphylaxis to NSAIDs, paracetamol, and lidocaine, as well as urticaria/angioedema-type reactions to NSAIDs and paracetamol (Table 2).

### 3.5. Drug Hypersensitivity Reactions to COVID-19 Vaccine

No hypersensitivity reactions were observed among the 23 patients who received COVID-19 vaccination (BNT162b2 [Pfizer–BioNTech, Mainz, Germany] or CoronaVac [Sinovac, Beijing, China]). As the patients had not been diagnosed with SM at the time of COVID-19 vaccination, they reported that they were not receiving any routine mastocytosis-directed treatment prior to vaccination.

## 4. Discussion

In this study, we evaluated the prevalence and clinical characteristics of DHRs in a well-characterized cohort of patients with systemic SM. A considerable proportion of patients experienced DHRs, most commonly in response to NSAIDs, paracetamol, and β-lactam antibiotics. In our cohort, many of these reactions presented as systemic and clinically significant events, with anaphylaxis often accompanied by hypotension being the most common clinical presentation.

One notable finding of our study is that drug-induced anaphylaxis represented the first and only clinical manifestation of SM in a subset of patients. None of these patients had a history of spontaneous anaphylaxis. Although this observation was identified in a limited number of patients, it suggests that clinicians should consider the possibility of an underlying mast cell disorder in individuals presenting with severe drug-induced anaphylaxis.

The occurrence and severity of drug-induced reactions in SM may be related to the increased mast cell burden and enhanced mediator releasability characteristic of this clonal disorder [10]. Several patients in our cohort exhibited a multiple-drug reactor phenotype, experiencing anaphylaxis to more than one pharmacological class. Although drug-induced anaphylaxis in SM has been reported previously, detailed class-specific data on reaction severity remain limited [7,11]. Our findings provide additional descriptive data suggesting that severe reactions may occur across multiple drug classes and highlight the importance of individualized, class-specific risk assessment in these patients.

### 4.1. NSAIDs and Paracetamol

NSAID hypersensitivity is a common cause of DHRs worldwide and is encountered relatively frequently in clinical practice. In the general population, NSAIDs account for approximately 21–25% of reported adverse drug events, highlighting their prominent role among drug-related reactions [12]. However, the prevalence of adverse reactions in mastocytosis patients is known to be higher, and due to concerns over severe anaphylaxis, avoidance of NSAIDs has historically been frequently recommended [11,13]. In the literature, the prevalence of NSAID hypersensitivity among patients with SM has been reported to be about 13% [14]. However, our cohort differed markedly in terms of reaction severity. While previous studies have reported predominantly mild, cutaneous reactions, most NSAID-related reactions in our patients were moderate-to-severe anaphylaxis [15].

In addition, paracetamol-associated anaphylaxis was observed in two patients in our cohort. However, both of these patients also had a history of NSAID reactions, which may suggest a possible cross-intolerance pattern rather than a specific hypersensitivity to paracetamol. Furthermore, given the very limited number of cases, this finding should be interpreted with caution and cannot support a definitive association between paracetamol and severe reactions in patients with mastocytosis. Therefore, this observation should be considered an isolated finding rather than evidence of a specific risk related to paracetamol use in this patient population.

Consistent with earlier reports, patients with NSAID hypersensitivity in our cohort predominantly had ISM or BMM [14]. Several patients also demonstrated reactions to more than one pharmacological class, suggesting a multiple-drug reactor phenotype. These observations indicate that patterns of drug hypersensitivity in mastocytosis may vary substantially between individuals and may not be limited to a single drug class.

Although mast cell burden has been proposed as a factor that may influence the clinical expression of hypersensitivity reactions [16], the relationship between mast cell burden and drug reactivity remains complex and was not statistically demonstrated in our cohort. Rather than blanket drug avoidance, these findings support an approach based on individualized risk stratification and supervised identification of safe therapeutic alternatives in patients with a history of drug hypersensitivity [11,17].

### 4.2. β-Lactam Antibiotics

Patients with SM have been suggested to experience antibiotic-induced hypersensitivity reactions more frequently than the general population [13]. In a comprehensive study investigating hypersensitivity reactions to antibiotics (HRA) in mastocytosis, the prevalence of HRA was reported as 14.2%, with the majority of reactions (74%) limited to cutaneous symptoms and a very low anaphylaxis rate of 0.8% [18]. Notably, that study found no significant differences in age, gender, atopic status, or baseline tryptase levels between mastocytosis patients with and without antibiotic hypersensitivity.

In contrast to previous reports describing predominantly mild cutaneous reactions to antibiotics in mastocytosis [18], all β-lactam reactions observed in our cohort presented as severe anaphylaxis, frequently associated with hypotension. Although patients who experienced these reactions appeared to have relatively elevated baseline tryptase levels, our analysis did not demonstrate a statistically significant difference in baseline tryptase levels between patients with and without DHRs. Therefore, our findings do not support a significant association between baseline tryptase levels and reaction severity [14,19].

Accordingly, the clinical relevance of the higher tryptase values observed in individual cases should be interpreted with caution. This observation may partly reflect the limited sample size of our cohort, and larger studies are required to better clarify the role of baseline tryptase levels in predicting antibiotic-induced anaphylaxis in patients with SM. Nevertheless, given the potential for severe reactions observed in some patients, careful clinical evaluation and individualized decision-making regarding antibiotic use remain advisable in patients with SM [17].

### 4.3. Quinolones

In our cohort, none of the patients who were exposed to quinolones reported hypersensitivity reactions. To further evaluate this tolerance clinically, oral provocation tests with fluoroquinolones were performed in three patients with a confirmed history of anaphylaxis to β-lactam antibiotics. All three patients tolerated quinolones during controlled challenges.

Given the limited number of tested patients, these findings should be interpreted with caution. Nevertheless, this observation may indicate that drug hypersensitivity in SM can be drug-specific rather than reflecting a generalized hyperreactive response to all potential mast cell—activating agents.

This clinical observation can also be considered in the context of recent molecular studies on the MRGPRX2. This receptor has been implicated in non—IgE-mediated mast cell activation and has been shown to be expressed in skin mast cells of patients with both cutaneous and ISM [20,21]. However, previous studies have reported that MRGPRX2 expression is not clearly associated with disease burden, baseline tryptase levels, or the severity of clinical symptoms [21]. This suggests that drug hypersensitivity in SM is often drug-specific rather than a manifestation of generalized hyperreactivity, and that increased receptor expression does not necessarily translate into clinical intolerance [19].

### 4.4. Anesthetics

In our cohort, reactions to local anesthetics were uncommon, with only a single drug reaction observed. This finding is generally consistent with previously published data. For example, a Spanish cohort study reported only a small number of mediator-release symptoms among 35 pregnant patients with ISM who received epidural or local anesthesia [22]. Similarly, a larger retrospective analysis demonstrated that confirmed local anesthetic allergy was identified in only 1.9% of 252 patients with mastocytosis [23]. Taken together, these findings suggest that hypersensitivity reactions to local anesthetics appear to be relatively infrequent in the mastocytosis population. However, the occurrence of a reaction in a patient with a history of multiple DHRs highlights the importance of careful individual risk assessment prior to any medical procedure.

With regard to general anesthesia, no hypersensitivity reactions were observed in patients who had undergone general anesthesia prior to the diagnosis of SM and without premedication. This observation is broadly consistent with the low perioperative reaction rates reported in larger cohort studies [22]. However, given the relatively small sample size of our cohort, these findings should be interpreted with caution and should not be considered evidence that general anesthesia is entirely risk-free in patients with SM. These observations may also suggest that perioperative risk in mastocytosis is heterogeneous and may vary according to individual clinical characteristics.

Overall, our findings suggest that hypersensitivity reactions to both local and general anesthetics may be uncommon in patients with SM. Nevertheless, the relatively small sample size of our cohort limits the ability to draw definitive conclusions, and therefore these findings should be interpreted cautiously. Our observations add to the limited body of evidence regarding perioperative drug tolerance in patients with mastocytosis. Further large-scale prospective studies are needed to better define the perioperative risk profile and optimize management strategies in this patient population.

The use of local and general anesthetics in patients with SM requires careful consideration because mast cell activation may predispose these patients to perioperative hypersensitivity reactions. Nevertheless, available evidence suggests that most patients with mastocytosis generally tolerate anesthetic agents well. Therefore, perioperative management in mastocytosis should primarily rely on individualized risk assessment based on the patient’s clinical history rather than the theoretical triggering potential of specific drugs. In addition, physical triggers such as friction, pressure, and stress may contribute to mast cell activation and should be minimized during the perioperative period. It is also important to recognize that intraoperative anaphylaxis may occasionally present solely as sudden hypotension without accompanying cutaneous manifestations. Accordingly, individualized premedication strategies and careful perioperative monitoring should be considered, particularly in patients at higher risk [24].

### 4.5. Radiocontrast Media

Careful management during RCM administration is recommended in patients with SM, as mast cell activation may predispose these patients to hypersensitivity reactions. In the general population, immediate hypersensitivity reactions to RCM are relatively uncommon, with an estimated incidence below 1% [25]. Although case reports and some clinical guidelines suggest that patients with mastocytosis may require particular attention during RCM exposure, robust evidence demonstrating an increased risk in this population remains limited [11,26,27].

In our cohort, one patient experienced anaphylaxis following RCM administration. However, the specific contrast agent involved could not be identified, and detailed clinical information regarding the reaction was limited. Given that only a single case was observed and the exact trigger remained uncertain, this observation should be interpreted with caution. Therefore, our data do not allow definitive conclusions regarding the overall risk of RCM reactions in patients with SM. Rather, this finding should be considered an isolated clinical observation. Notably, the patient who experienced the reaction had a history of multiple severe drug reactions, including hypersensitivity to NSAIDs, paracetamol, and lidocaine. This pattern may reflect a broader predisposition to drug hypersensitivity in this individual and could be consistent with a phenotype characterized by increased mast cell reactivity.

Because robust controlled studies evaluating optimal RCM management in mastocytosis remain scarce [11], current clinical practice largely relies on risk stratification and established guideline recommendations [17]. Premedication and careful monitoring are generally advised, particularly in patients with prior hypersensitivity reactions or other high-risk clinical features. Nevertheless, larger prospective and systematic studies are needed to better clarify the true risk of RCM reactions in patients with mastocytosis.

### 4.6. Vaccination

Importantly, no hypersensitivity reactions to COVID-19 vaccines were observed in our cohort, despite the absence of premedication and the fact that patients had not yet been diagnosed with SM at the time of vaccination. Evidence on vaccination in mastocytosis is largely derived from retrospective studies, which generally suggest that most adult patients tolerate vaccines well, although adverse reactions have occasionally been reported [28,29,30]. Previous studies evaluating COVID-19 vaccination in patients with mastocytosis have also demonstrated generally good tolerance, with most reported reactions being mild.

Although the number of vaccinated patients in our cohort was limited, this observation is consistent with previous reports indicating that vaccination is generally well tolerated in adult patients with mastocytosis. Therefore, our findings are in line with current recommendations suggesting that vaccination should not be systematically avoided in patients with SM. Instead, vaccination may be administered following individualized risk assessment, with premedication considered when clinically indicated [31,32].

This study provides real-world clinical data on DHRs in patients with SM evaluated in a tertiary referral center and contributes to the limited literature on this clinically important topic. SM is characterized by clonal mast cell proliferation and increased mast cell mediator release, which may predispose patients to hypersensitivity reactions to various triggers, including medications. Therefore, recognition and appropriate evaluation of drug-related reactions in this patient population are essential for both diagnostic assessment and safe therapeutic management.

In our cohort, drug hypersensitivity was confirmed in a minority of patients through positive diagnostic testing, whereas in the remaining cases the diagnosis was established based on a compatible clinical history and medical record documentation. This observation reflects the diagnostic challenges frequently encountered in patients with severe reaction histories. In clinical practice, confirmatory testing with the culprit drug is not always feasible because of safety concerns, particularly in patients with a history of severe systemic reactions. In such cases, allergological evaluation with alternative medications may provide clinically relevant information and help identify safe therapeutic options for future treatments.

Several limitations should be considered when interpreting our findings. First, the relatively small sample size may limit the statistical power of the analyses and reduce the ability to detect potential associations between clinical variables and DHRs. Therefore, the absence of statistically significant differences should not be interpreted as evidence of the absence of a true association. Second, the retrospective design of the study introduces inherent limitations related to data collection and documentation. Moreover, some drug reactions occurred several years before the diagnosis of SM, and therefore recall bias related to patient-reported history cannot be completely excluded.

Despite these limitations, SM is a rare disorder, and studies specifically addressing DHRs in this population remain limited. Our findings provide clinically relevant insights into the spectrum of DHRs observed in patients with SM in routine clinical practice. Increased awareness of drug-induced reactions in this patient population is important, as such reactions may reflect increased mast cell reactivity and mediator release. Careful evaluation of suspected drug reactions, including detailed clinical history, appropriate allergological testing, and identification of safe alternative medications, is essential to optimize patient safety and clinical management.

## 5. Conclusions

Overall, our study provides real-world clinical insights into DHRs in patients with SM. While many patients with SM tolerated medications without complications, a subset of patients in our cohort experienced severe hypersensitivity reactions, often involving multiple drug classes, particularly NSAIDs and beta-lactam antibiotics. In addition, drug allergy may represent the first and only clinical manifestation of SM in some patients. Therefore, measurement of baseline serum tryptase is of critical importance in patients presenting with drug-induced anaphylaxis. These findings highlight the importance of careful clinical evaluation, individualized risk assessment, and identification of safe alternative medications rather than generalized drug avoidance. Future prospective and multicenter studies are needed to better define the clinical characteristics and potential risk factors associated with DHRs in patients with SM.

## Figures and Tables

**Table 1:** Comparative Table of Systemic Mastocytosis Patient Groups.

Variables	Total Cohort (*n* = 34)	DHR (−) (*n* = 24)	DHR (+) (*n* = 10)	Anaphylaxis as First/Only Symptom (*n* = 5)
Age at diagnosis (Years)	48.6 ± 13.3 (Mean)	49.9 ± 14.3 (Mean)	44.4 ± 12.6 (Mean)	36 (Median)
Gender (Male)	18 (53%)	13 (54.1%)	5 (50%)	1 (20%)
Baseline Tryptase (µg/L) (Median)	50.25	57.40	41.85	34.60
SM Subtypes				
BMM	8 (23.5%)	4 (16.6%)	4 (40%)	2 (40%)
ISM	17 (50%)	12 (50%)	5 (50%)	2 (40%)
SSM	1 (3%)	1 (4.1%)	0	0
ASM	4 (11.7%)	4 (16.6%)	0	0
SM-AHN	4 (11.7%)	3 (12.5%)	1 (10%)	1 (20%)

Abbreviations: ASM, aggressive systemic mastocytosis; BMM, bone marrow mastocytosis; DHR, drug hypersensitivity reaction; ISM, indolent systemic mastocytosis; SM, systemic mastocytosis; SM-AHN, systemic mastocytosis with an associated hematologic neoplasm; SSM, smoldering systemic mastocytosis.

**Table 2:** Demographic, Clinical, and Laboratory Characteristics of the Patients who had a history of drug, radiocontrast media, and COVID-19 vaccine induced anaphylactic reactions.

Patient No	Gender/ Age	SM Type	BT (µg/L) (<14)	Total IgE (kU/L) (<100)	Specific IgE (Penicillin Determinant)	Reaction Type	NSAID	Paracetamol	Beta- Lactam	RCM	Local Anesthesia	Time Interval (min)	Symptom	Treatment	Skin Tests/DPTs for DHRs
P 1	M/52	BMM	50.00	-		Grade 4 Anaphylaxis	Propyphenazone	+	-	#	-	15	Generalized pruritus Shortness of breath Loss of consciousness Diarrhea Tachycardia Dyspnea Hypotension Cardiac arrest	Defibrillation Epinephrine 0.3 mg IM Pheniramine Methylprednisolone	No
P 2	F/30	BMM	46.20	31.9		Grade 3 Anaphylaxis	Flurbiprofen Ibuprofen Diclofenac	-	-	#	-	60–120 240 45	Shortness of breath, shivering Blurred vision, vomiting Dizziness, Visual loss, Vomiting, Transient loss of consciousness, Facial flushing Hypotension Tachycardia Shortness of breath	1: Resolved spontaneously after 15 min 2.3: Pheniramine	Paracetamol oral DPT: No reaction Meloxicam oral DPT: No reaction
P 3	M/53	ISM	200.00	4.6	Penicilloyl G: Negative Penicilloyl V: Negative Ampicillin: Negative	Grade 3 Anaphylaxis	-	-	Ampicillin	#	-	360	Hypotension, Nausea, Vomiting, Shortness of breath	One day admission to the ICU	No
P 4	M/36	BMM	126.00	63.2	Penicilloyl G: Positive Penicilloyl V: Positive Amoxicillin: Negative	Grade 3 Anaphylaxis	-	-	Amoxicillin	-	-	45	Nausea Shortness of breath Dizziness Itching and redness on the hands Syncope Chest pain Fecal incontinence Hypotension	Pheniramine	DAP test: + Ciprofloxacin oral DPT: No reaction Clarithromycin oral DPT: No reaction
P 5	F/53	ISM	37.50	711		1–2: Grade 3 3: Grade 2 4: Grade 2 Anaphylaxis	Naproxen	+	-	+	Lidocaine	20 20 15 60	Itching, Redness Hypotension, Syncope Redness, Rash, Dizziness Redness, Itching, Vomiting	Pheniramine	Skin Prick-ID test: Negative for fentanyl, propofol, latex, tramadol and rocuronium
P 6	M/42	BMM	19.00	128		Urticaria Angioedema	Indomethacin	-	-	-	-	240	Generalized urticaria and edema in the genital area	Pheniramine	No
P 7	F/43	ISM	20.40	441	Penicilloyl G: Negative	Grade 3 Anaphylaxis	-	-	Amoxicillin	#	-	30	Urticaria, shortness of breath, palpitations Altered mental status Desaturation	Methylprednisolone Pheniramine	Cefuroxime, Ceftriaxone, Penicillin Ampicillin–Sulbactam Skin Prick-ID: No reaction Ceftriaxone intramuscular DPT: No reaction Cefuroxime oral DPT: No reaction Levofloxacin oral DPT: No reaction
P 8	F/70	SM-AHN	23.30	11.9	Penicilloyl G: Negative Penicilloyl V: Positive	Grade 3 Anaphylaxis	-	-	Amoxicillin	#	-	30	Facial flushing, Generalized itching, Hypotension, Syncope	Epinephrine 0.3 mg IM	Meloxicam oral DPT: No reaction Clarithromycin, Metronidazole, Clindamycin oral DPT: No reaction
P 9	M/33	ISM	111.00	19.4		Grade 2 Anaphylaxis	Naproxen	-	-	-	-	10	Facial flushing, Shortness of breath, and Tachycardia	Pheniramine	Meloxicam oral DPT: No reaction
P 10	F/32	ISM	34.6	131	Penicilloyl G: Negative Penicilloyl V: Negative	1: Grade 3 2: Grade 2 Anaphylaxis	Ibuprofen Propyphenazone	-	Ampicillin	-	-	10 30	Facial redness, Dizziness, Fatigue, Palpitations, Shortness of breath, Hypotension, Tachycardia Facial redness, itching, Shortness of breath,	Epinephrine 0.3 mg IM	Ciprofloxacin oral DPT: No reaction

Abbreviations: BMM, bone marrow mastocytosis; BT, baseline tryptase; DAP, determinants of allergic penicillin; DHR, drug hypersensitivity reaction; DPT, drug provocation test; ID, intradermal; IM, intramuscular; ISM, indolent systemic mastocytosis; Min, minutes; NSAID, nonsteroidal anti-inflammatory drug; RCM, radiocontrast media; SM-AHN, systemic mastocytosis with associated hematologic neoplasm; +, reaction; -, no reaction; #, no use.

## Data Availability

The data presented in this study are available on request from the corresponding author. The data are not publicly available due to privacy restrictions.

## Abbreviations

The following abbreviations are used in this manuscript:

SM	Systemic Mastocytosis
DHR	Drug Hypersensitivity Reaction
NSAIDs	Nonsteroidal Anti-inflammatory Drugs
RCM	Radiocontrast Media
WHO	World Health Organization
BMM	Bone Marrow Mastocytosis
ISM	Indolent Systemic Mastocytosis
SSM	Smoldering Systemic Mastocytosis
ASM	Aggressive Systemic Mastocytosis
SM-AHN	Systemic Mastocytosis with an Associated Hematologic Neoplasm
MRGPRX2	Mas-related G Protein—Coupled Receptor X2
SPT	Skin Prick Test
IDT	Intradermal Test
DPT	Drug Provocation Tests
DAP	Determinants of Allergic Penicillin
